# The Standard Dictionary

**Published:** 1894-07

**Authors:** 


THE STANDARD DICTIONARY of Funk & Wagnalls, heretofore reviewed in these pages, has the following points not noticed in the review.

If a word in the vocabulary has more than one pronunciation, the one first given is that preferred by the Standard; those that follow are from other dictionaries. The Roman numeral XIII refers to the Appendix for pronunciations preferred by the Advisory Committee and other authorities. (See *Introductory*, Vol. I, p. 9.)

In the spelling of names in geography, the decisions of the United States Board on Geographic Names have been followed, the Committee of that Board kindly consenting to pass upon all words that might from time to time be submitted to them. These spellings and pronunciations of geographic names will be given in the Appendix of the Dictionary.

That there is a drift conservative yet real toward the simpler forms of spelling has been recognized throughout the work. In all words fully Anglicized "e" has been preferred to the diphthongs "æ" and "œ," as in fe[œ]tus, home[œ]opathy, e[æ]sthetics. In cases, however, where diphthongal forms are still largely or prevailingly used in current literature, the two forms have been bracketed in vocabulary place; as, esthetics / æsthetics }. When English and American usage differs, as in the spelling of honor (honour), favor (favour), the simpler form has been given the preference, but the English form has also been given a vocabulary place. The use of the dieresis has been discarded, as there seemed to be no sufficient reason for indicating, in ordinary writing and printing, the pronunciation of words used, as cooperative (coöperative), zoology (zoölgy). Vocabulary places have been given to the three thousand five hundred words to which the American Philological Association and the American Spelling Reform Association recommend the immediate application of the principles of spelling reform—principles that have been adopted also by the Philological Society of England.

